# Carbapenem-resistant gram-negative bacteria in Germany: incidence and distribution among specific infections and mortality: an epidemiological analysis using real-world data

**DOI:** 10.1007/s15010-022-01843-6

**Published:** 2022-05-31

**Authors:** Michael H. Wilke, Birgit Preisendörfer, Anna Seiffert, Maria Kleppisch, Caroline Schweizer, Stephan Rauchensteiner

**Affiliations:** 1 Medical School Hamburg (MSH), Am Kaiserkai 1, 20456 Hamburg, Germany; 2 Inspiring-Health GmbH, Waldmeisterstrasse 72, 80935 Munich, Germany; 3 Gesundheitsforen Leipzig GmbH, Hainstraße 16, 04109 Leipzig, Germany; 4 Health Technology Assessment and Outcomes Research (HTA&OR), Health and Value Germany, Pfizer Pharma GmbH, Linkstraße 10, 10785 Berlin, Germany; 5 Hospital Business Unit Germany, Pfizer Pharma GmbH, Linkstraße 10, 10785 Berlin, Germany

**Keywords:** Carbapenem-resistant gram-negative bacteria, Epidemiology, Germany, Claims data, Ceftazidime Avibactam (CAZ/AVI), Infections, Mortality, 4MRGN

## Abstract

**Purpose:**

Infections with carbapenem-resistant gram-negative bacteria (in Germany classified as 4MRGN) are a growing threat in clinical care. This study was undertaken to understand the overall burden of 4MRGN infections in Germany in the context of a Health Technology Appraisal (HTA) for Ceftazidime/Avibactam (CAZ/AVI). Besides, the incidences mortality was an endpoint of interest.

**Methods:**

To assess infections with carbapenem-resistant gram-negative bacteria and related mortality, three different data sources have been used. From the German statistics office (DESTATIS) data have been retrieved to obtain the overall frequency these pathogens. Via two other databases, the German analysis database (DADB) and a Benchmarking of > 200 hospitals in a representative sample (BM-DB), the distribution of the infections and the mortality have been analyzed.

**Results:**

DESTATIS data showed a total of 11,863 carbapenem-resistant gram-negative bacteria codings, of which 10,348 represent infections and 1515 carriers. The most frequent infections were complicated urinary tract infections (cUTI) (*n *= 2,337), followed by pneumonia (*n* = 1006) and intra-abdominal infections (*n* = 730). A considerable amount of patients had multiple infections in one hospital episode (*n* = 1258). In-hospital mortality was 18.6% in DADB and 14.3% in the BM-DB population, respectively. In cases with additional bloodstream infections, DADB mortality was correspondingly higher at 33.0%. DADB data showed an incremental mortality increase of 5.7% after 30 days and 10.0% after 90 days resulting in a cumulative 90 day mortality of 34.3%.

**Conclusions:**

Infections with carbapenem-resistant gram-negative bacteria are still rare (6.8–12.4 per 100,000) but show a significant increase in mortality compared to infections with more sensitive pathogens. Using different data sources allowed obtaining a realistic picture.

**Supplementary Information:**

The online version contains supplementary material available at 10.1007/s15010-022-01843-6.

## Introduction

Infections with carbapenem-resistant gram-negative bacteria offer only limited treatment options (LTO) have worse outcomes and are growing in number in Germany [1–5]. For this reason, the subject is of great interest to the scientific community. CAZ/AVI is a combination of Ceftazidime (cephalosporin) and Avibactam (Beta-lactamase inhibitor) [6, 7]. CAZ/AVI is a reserve antibiotic indicated for the use in complicated Intra-Abdominal Infections (cIAI), complicated Urinary Tract Infections (cUTI), Hospital-Acquired and Ventilator-Associated Pneumonia (HAP/VAP) and in Bacteremia associated with one of these indications. Its label also covers any other infection with LTO where a carbapenem-resistant gram-negative bacteria is causative or likely to be causative. It is active against most carbapenem-resistant gram-negative bacteria pathogens except *Acinetobacter baumannii*.

The primary objective of this research was obtaining realistic numbers of patients / hospital cases for Germany with infections caused by a carbapenem-resistant gram-negative bacteria which are in the label of CAZ/AVI in the context of a HTA dossier. Secondary objectives were gaining an overview on mortality as well as on the health economic impact of these infection (the latter will not be part of this publication).

## Materials and methods

In Germany infections and carrier detections of carbapenem-resistant gram-negative bacteria have to be reported to the German Robert-Koch Institute (RKI) [8]. In Germany the carbapenem-resistant gram-negative bacteria are referred as 4MRGN, i.e., as bacteria with resistance against four major antibiotic classes, including carbapenem resistance. However, this reporting does not compromise cases with pseudomonas aeruginosa. Acinetobacter where CAZ/AVI is not effective is reported. Although reporting is mandatory in Germany, RKI suspects a certain amount of underreporting [8]. Since RKI reporting does not cover all pathogens of interest and does not elaborate on the underlying infections, we chose to collect administrative claims data based on billing information of the Statutory Health Insurance (SHI). The data are frequently used and have good quality in Germany [9–11]. The infections in label of CAZ/AVI are complex infections. Before using claims data these infections had to be coded in ICD-10. For this research different data sources have been used. From the German statistics office (DESTATIS) overall coding frequencies of carbapenem-resistant gram-negative bacteria were retrieved. The data do not differentiate between infections and colonizations and complex combinations of codes cannot be obtained. To gain insight in the infections two more databases were queried. DADB contains data of app. 2.8 million SHI insured individuals and can provide longitudinal information, such as 30- and 90 day mortality and has frequently been used in analyses [12, 13]. The BM-DB is a database used for hospital benchmarking purposes and contains data from 300 hospitals with more than 4 million inpatient episodes and has also been the basis for claims data-based analyses [14]. Both databases provide anonymized patient-level data of all diagnoses, procedures that have been coded during a hospital stay. We provide a tabular comparison of the databases as supplementary material.

To identify the patients of interest, the coded diagnoses were used. Primary and secondary diagnoses in Germany are coded according to the 10th revision of the International Classification of diseases, German modification (ICD-10 GM). This was done by using recent guidelines and publications for cUTI, cIAI, HAP/VAP, Bacteremia, sepsis, and LTO infections and abstracting the ICD-10 codes based on the definitions [15–25]. By connecting several ICD-10 GM codes it was possible to determine the presence of a complicated infection. A full list of the ICD-10 GM codes and combinations used for this research is shown in the supplemental tables.

The calculation of potential patient numbers was done in several steps as outlined in diagram no. 1
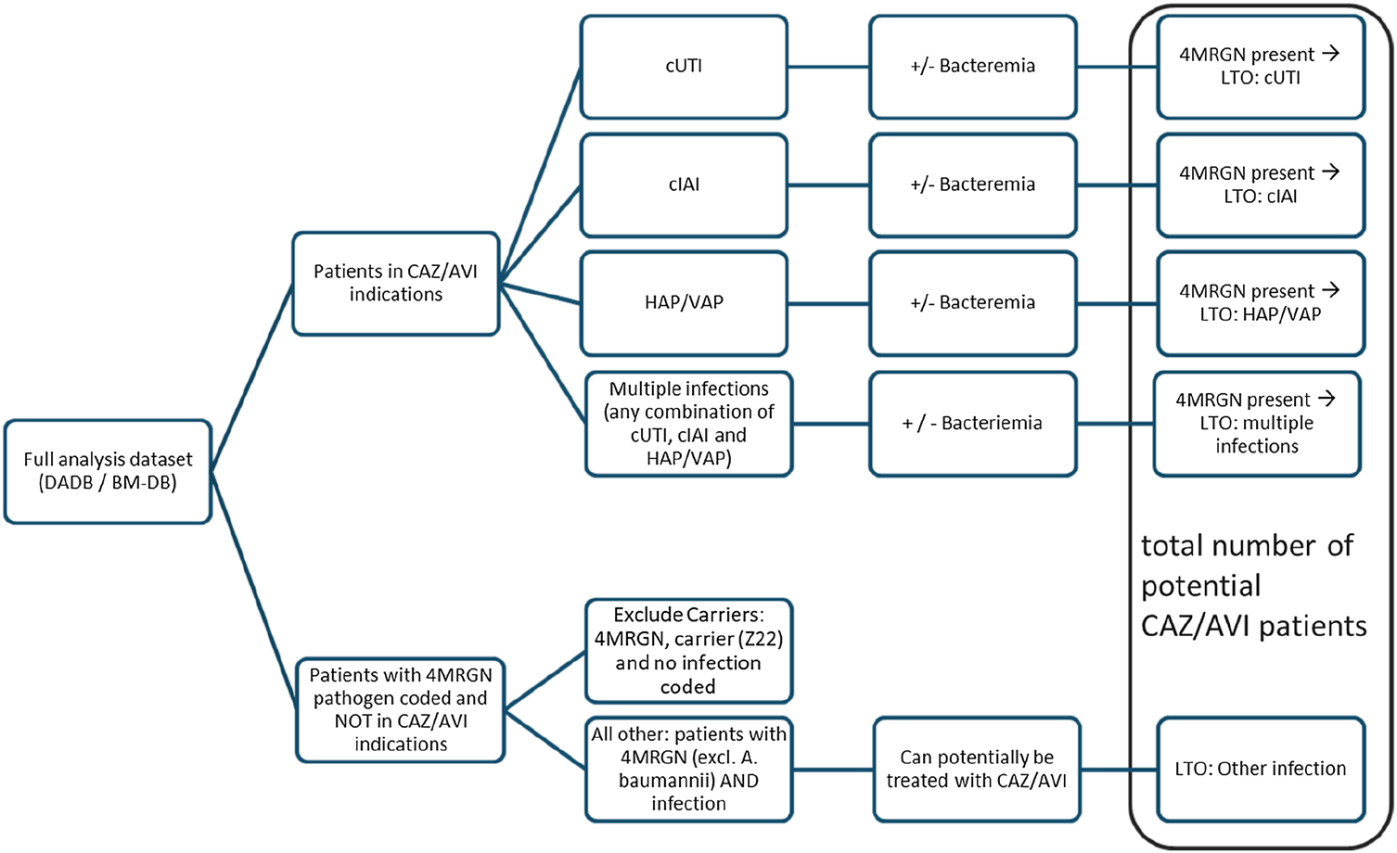


The patient numbers retrieved from the databases were then extrapolated for Germany. 73 million German citizens (90%) are insured in the SHI [26]. A first extrapolation was done to calculate the incidence of infections caused by carbapenem-resistant gram-negative bacteria in the total SHI population. In the next step the incidence in the total population was extrapolated. In DADB patients were extrapolated in age and gender groups to match the distribution in the total SHI population. The data from BM-DB were extrapolated using the total number of carbapenem-resistant gram-negative bacteria and the total number of hospitalizations from DESTATIS. In both cases results were calculated for the SHI and the total German population. For all patient groups the in-hospital mortality was calculated in both databases. From DADB 30- and 90 day mortality have been additionally retrieved.

## Results

For 2019 DESTATIS reported a total of 12,737 inpatient cases with a coding of any carbapenem-resistant gram-negative bacteria. The distribution of the pathogens is shown in (Table 1). After deducting of cases with *Acinetobacter baumannii* group (*n* = 874), 11,683 cases had a pathogen coded where CAZ/AVI is potentially active. This number was used to do further extrapolations from the BM-DB, while the numbers from DADB have been extrapolated using the age and gender tables. This method has been used before on this specific database [12, 13]. With the case-level data the distribution of infections and carriers among the indication of CAZ/AVI was derived from the databases and then extrapolated.

**Table 1:** Overview coded pathogens

4MRGN codings, reported by DeStatis		
		Destatis
ICD-10	Pathogen	cases (*n* =)
	Total enterobacterales	**5.790**
U81.40	Escherichia coli	1.464
U81.41	Klebsiella pneumoniae	1744
U81.42	Klebsiella oxytoca	209
U81.43	Other Klebsiella	350
U81.44	Enterobacter cloacae complex	827
U81.45	Citrobacter freundii complex	349
U81.46	Serratia marcescens	131
U81.47	Proteus mirabilis	134
U81.48	Other Enterobacterales	582
U81.50	Pseudomonas aeruginosa	**6.073**
U81.51	Acinetobacter baumannii group	**874**
	Grand total	**12.737**
	Total w/o A. baumannii	**11.863**

Distribution of coded pathogens from DESTATIS, by ICD-10

The extrapolation from DADB resulted in a total of 7196 cases, while the extrapolation from BM-DB resulted in consequently in 11,683 cases. While in BM-DB other infections was the biggest group (n = 818, extrapolated *n* = 5,017, 42.3% of all cases), in DADB the biggest group were multiple infections, i.e., combinations of HAP/VAP, cUTI, and cIAI (*n* = 56, extrapolated 1,644, 22.8% of all cases). The results from both databases together with the extrapolations on Germany for SHI and total population are displayed in (Table 2).

**Table 2:** Case numbers and extrapolations

4MRGN analysis 2019	DADB	Extrapolation 1		BM-DB	Extrapolation 2	
Infection entities w 4MRGN	Cases	SHI population	Total population	Cases	SHI population	Total population
cIAI total	**15**	**387**	**440**	**119**	**640**	**730**
w/o bacteremia	6	155	176	95	511	583
With bacteremia	9	232	264	24	129	147
cUTI total	**50**	**1.289**	**1.469**	**381**	**2.052**	**2.337**
w/o bacteremia	39	1005	1145	309	1.664	1.895
With bacteremia	11	284	323	72	388	442
HAP/VAP total	**23**	**593**	**676**	**164**	**883**	**1.006**
w/o bacteremia	16	412	470	127	684	779
With bacteremia	7	180	206	37	199	227
Multiple infections (separate counting)	**56**	**1.444**	**1.644**	**205**	**1.104**	**1.258**
w/o bacteremia	31	799	910	117	630	718
With bacteremia	25	645	734	88	474	540
Other infections total	**48**	**1.237**	**1.410**	**818**	**4.404**	**5.017**
w/o bacteremia	44	1.134	1.292	737	3.968	4.520
With bacteremia	4	103	117	81	436	497
Carriers (no infection coded)	**54**	**1.366**	**1.557**	**247**	**1.330**	**1.515**

Total	246	6.316	7.196	1.934	10.413	11.863
Total w/o Carrier	**192**	**4.950**	**5.639**	**1.687**	**9.083**	**10.348**

Case numbers and extrapolations from DADB and BM-DB

No. of DADB SHI patients in 2019: 2.831.295

No. of SHI patients in 2019: 72.995.384 (Extrapolation factor: 25, 78)

Total population in 2019: 83.166.711 (Extrapolation factor: 29, 37)

BM-DB Total no. of cases in BM-DB in 2019: 4.111.603; DESTATIS: total no. of cases 18.823.421; DESTATIS: total no. of 4MRGN 11.863, Share SHI of total population = 8777%

BM-DB: hospital Benchmark Data base INMED

*DADB* German Analysis Database for Evaluation and Health Services Research, *SHI* Statutory Health Insurance

Mortality analyses showed a total of 14.3% in BM-DB (277/1,934) and 18.6% in DADB (39/210). From DADB it was possible to follow the patients up to 90 days after hospital discharge. The cumulative mortality went up to 24.3% after 30 days and 34.3% after 90 days. The data show—while on a small sample from DADB—that mortality significantly increases if it is observed in a 90 day window. The complete overview on mortalities is provided in (Table 3).

**Table 3:** Mortality

Infection/Carrier	4MRGN Mortality BM-DB 2019			4MRGN Mortality DADB						
	All inpatient cases			All inpatient cases			30 days after discharge		90 days after discharge	
	Inpatient cases with 4MRGN (*n* =)	No. of cases died (*n* =)	In-hospital mortality (%)	Inpatient cases with 4MRGN (*n* =)	No. of cases died (*n *=)	In-hospital mortality (%)	Additional no. of cases died (*n* =)	30-day cu-mulative mortality (%)	Additional no. of cases died (*n* =)	90-day cu-mulative mortality (%)
cIAI	95	9	9.5	6	2	33.3	0	33.3	0	33.3
cIAI + bacteremia	24	7	29.2	9	2	22.2	0	22.2	1	33.3
cUTI	309	38	12.3	36	6	16.7	1	19.4	4	30.6
cUTI + bacteremia	72	14	19.4	6	2	33.3	1	50.0	1	66.7
HAPVAP	127	28	22.0	16	6	37.5	1	43.8	2	56.3
HAPVAP + bacteremia	37	19	51.4	7	2	28.6	0	28.6	0	28.6
Multiple infections	117	32	27.4	31	10	32.3	4	45.2	5	61.3
Multiple infections + bacteremia	88	35	39.8	23	6	26.1	2	34.8	5	56.5
LTO: Other infection	737	69	9.4	33	3	9.1	2	15.2	2	21.2
LTO: Other infection + bacteremia	81	17	21.0	4	0	0.0	1	25.0	1	50.0
Carrier	247	9	3.6	42	–	–	–	–		–
Total	**1.934**	**277**	**14.3**	**210**	**39**	**18.6**	**12**	**24.3**	**21**	**34.3**

Mortality analysis and comparison

## Discussion

This study was—to our knowledge—the first study that extrapolated the total burden of carbapenem-resistant gram-negative bacteria caused infections in Germany using different data sources. While DADB was used several times before to extrapolate certain patient volumes in Germany, we used additionally BM-DB with a much larger sample of inpatients and anonymized case-level data available.

One main finding of this study shows that numbers of carbapenem-resistant gram-negative bacteria infections are higher than the numbers of isolates reported by RKI and by the ARS system [3, 8]. One reason is pseudomonas aeruginosa missing in RKI data [8]. The direct comparison of pathogens that have to be reported shows rates of 79.9% in Enterobacterales and 81.4% in *A. baumannii* complex. On pathogen level numbers vary between 42.5% (*Proteus mirabilis*) and even 121% (other *Klebsiella*). Moreover, data sources are different. We analyzed data from claims using ICD-10 coding, while RKI receives data from laboratories if those find carbapenem-resistant gram-negative bacteria isolates or send isolates for differentiated analyses which are corrected by duplicates to find the case numbers. Looking at the overall match the pathogens which are reported by RKI and coded in ICD-10 match quite good, with a “lack” of 20% in RKI data. The comparison of the data is shown in (Table 4).

**Table 4:** Comparison

4MRGN data reporting 2019, Comparison DeStatis-RKI				
		Destatis	RKI	Portion reported
ICD-10	Pathogen	Cases (*n* =)	Cases (*n* =)	In %
	Total Enterobacterales	**5.790**	**4.614**	**79.7%**
U81.40	Escherichia coli	1.464	936	63.9%
U81.41	Klebsiella pneumoniae	1.744	1.648	94.5%
U81.42	Klebsiella oxytoca	209	142	67.9%
U81.43	Other Klebsiella	350	424	121.1%
U81.44	Enterobacter cloacae complex	827	702	84.9%
U81.45	Citrobacter freundii complex	349	242	69.3%
U81.46	Serratia marcescens	131	107	81.7%
U81.47	Proteus mirabilis	134	57	42.5%
U81.48	Other Enterobacterales	582	356	61.2%
U81.50	Pseudomonas aeruginosa	**6.073**	**–**	**–**
U81.51	Acinetobacter baumannii group	**874**	**711**	**81.4%**

Comparison of 4MRGN codings DESTATIS and reporting RKI

Comparing the extrapolations from DADB and BM-DB we see different results. While DADB results into 7,196 cases, including carriers, the total number in BM-DB is—by nature of the extrapolation—congruent with the DESTATIS data. This difference can have different reasons. First of all, DADB does extrapolation on relatively low patient numbers. In groups where we found only two patients an error of 1 or 2 patients in the representability already leads to difference of 25 or 50 patients in the extrapolation. Moreover, infections or carriers of carbapenem-resistant gram-negative bacteria are generally rare. In DADB they account for 1.8% of all cases that have any infection in label of CAZ/AVI (245/13,795) and only for 0.008% of all patients (245/2,831,295). The extrapolation of very rare events bears the risk of error. In BM-DB cases with carbapenem-resistant gram-negative bacteria account for 0.3% of all cases in label of CAZ/AVI (1,934/216,846). In total they make 0.05% of all cases in BM-DB (1,934/4,111,603). The fundamental difference of the two databases is that DADB contains data of insured people and BM-DB is already a selection of inpatient cases. The total no. of inpatient cases was 19.4 Mio. In 2019 [27], consecutively BM-DB holds 21.2% of the total cases in Germany. In DADB the total no. of inpatient cases was 311,000 which is 1.6% of all inpatient cases in Germany.

Case numbers derived from coded data can be skewed by the fact that some codes are eventually relevant for billing but not clinically. Although all datasets undergo electronic plausibility checks in the insurance, 12.5% of all inpatient cases are audited by the medical service of the SHI [28]. The data quality can be considered good, but numbers can be higher than in reality due to upcoding. In Germany coded data have a direct connection between a primary code (e.g., infection) and a directly related secondary code (e.g., the resistance). Therefore, the German data allow a good distinction between colonization and infection as long as the medical record was appropriately interpreted. In general it is not easy to obtain reliable data on colonization vs. infection on a national level [29].

Mortality in-hospital was 14.3% in BM-DB and 18.6% in DADB, respectively. The 30-day all-cause mortality measured in DADB summed up to 24.3% and the 90-day mortality to 34.3%. Other studies report similar results. An analysis which yielded to develop a score reported 18 and 15% crude mortality and pooled data of 30 day mortality of 31.3% [30]. Other studies show 19% in-hospital mortality and 17.5% 30 day mortality in cIAI [31, 32]. The results from this real-world data analysis are therefore pretty much in line with those of other authors who derived results from retrospective chart reviews. One limitation is that mortality was measured unadjusted as all-cause mortality. This is often the case when routine data are used, as specific adjustments need broad clinical datasets.

In contrary to other studies that use real-world data for estimates of carbapenem-resistant gram-negative bacteria rates, we spend a lot of time to define the entities which are in the label of CAZ/AVI. By doing this we got a good overview not only of the totals but also of the distribution of the carbapenem-resistant gram-negative bacteria infections over the various indications. With this approach we were also able to identify the potential number of carriers who do not have an active infection. This number is similar in DADB and BM-DB extrapolation and is roughly 1,500 cases in 2019. DESTATIS only reports cases with carbapenem-resistant gram-negative bacteria and the code for carrier present. As this includes patients with active infections that carry the pathogen either on admission or on discharge, the number of 5,230 carriers in conjunction with a carbapenem-resistant gram-negative bacteria ICD-10 code is probably too high.

For a sound estimate of potential patient numbers in the given indication it has to be taken into account that CAZ/AVI is not active against Metallo-Betalactamases (MBL). The most recent RKI analyses counted app. 30% MBL in enterobacterales [33]. In pseudomonas spp. MBL are the most frequent carbapenamases. The numbers could be less than 50% of the observed infections with carbapenem-resistant gram-negative bacteria.

## Conclusion

The study on carbapenem-resistant gram-negative bacteria infections in Germany using two different databases and approaches for extrapolation revealed a number between 7196 and 11,683 cases in Germany with approximately 1,500 carriers of 4MRGN that have no active infections. The potential number of hospital cases for CAZ/AVI in Germany is between 5639 (DADB) and 10,348 (BM-DB) of which a certain number has to be deducted due to MBL present. The mortality in the infections with carbapenem-resistant gram-negative bacteria is high. 90-day mortality is 34.3%. The data underpin the need for effective antibiotic compounds in these infections, while the total amount of cases is still low (6.8–12.4 per 100,000).

## Supplementary Information

Below is the link to the electronic supplementary material.

Supplementary file1 (XLSX 24 KB)
